# Proteomic Analysis in Seminal Plasma of Fertile Donors and Infertile Patients with Sperm DNA Fragmentation

**DOI:** 10.3390/ijms21145046

**Published:** 2020-07-17

**Authors:** Alba Fernandez-Encinas, Agustín García-Peiró, Javier del Rey, Jordi Ribas-Maynou, Carlos Abad, Maria José Amengual, Elena Prada, Joaquima Navarro, Jordi Benet

**Affiliations:** 1 Departament de Biologia Cel·lular, Fisiologia i Immunologia, Universitat Autònoma de Barcelona, 08193 Bellaterra, Spain; alba.fernandeze@e-campus.uab.cat (A.F.-E.); Javier.DelRey@uab.cat (J.d.R.); j.ribas87@gmail.com (J.R.-M.); joaquima.navarro@uab.cat (J.N.); 2 Centro de Infertilidad Masculina y Análisis de Barcelona (CIMAB), 08192 Sant Quirze del Vallès, Spain; agusti.garcia@uab.cat; 3 Servei d’Urologia, Corporació Sanitària Parc Taulí, Sabadell, Institut Universitari Parc Taulí—UAB, 08208 Sabadell, Spain; cabad@tauli.cat; 4 UDIAT, Centre Diagnòstic, Corporació Sanitària Parc Taulí, Sabadell, Institut Universitari Parc Taulí—UAB, 08208 Sabadell, Spain; mjamengual@tauli.cat; 5 Servei de Ginecologia, Hospital Universitari Mútua de Terrassa, 08221 Terrassa, Spain; eprada@mutuaterrassa.es

**Keywords:** seminal plasma, 2D-DIGE, sperm DNA fragmentation, biomarkers, male infertility

## Abstract

Seminal plasma proteomics studies could represent a new approach for the determination of molecular elements driving male infertility, resulting in a better male infertility characterization. The aim of this study is to investigate proteomic differences in seminal plasma samples from fertile and infertile individuals. For that, semen samples were selected according to semen analysis, clinical pathology, and values of sperm DNA fragmentation (alkaline and neutral Comet assay and Sperm Chromatin Dispersion test). A total of 24 seminal plasma samples classified in four groups were processed: fertile donors (FD), recurrent miscarriage patients (RM), asthenoteratozoospermic patients (ATZ), and asthenoteratozoospermic patients with varicocele (ATZ-VAR). Results obtained by 2D-differential gel electrophoresis (2D-DIGE) revealed 26 spots significantly increased in fertile donors when compared to patient groups. Also, eight spots in the ATZ group and two in the ATZ-VAR group were decreased compared to the other groups. Twenty-eight proteins were identified by mass spectrometry (MS), most of them involved in metabolic and cellular processes and with a catalytic or binding function. Protein–protein interactions through Search Tool for the Retrieval of Interacting Genes/Proteins (STRING) tool suggest that a large part of them were associated with each other. Furthermore, most of them were associated with ubiquitin C, indicating that it could play an important regulation role, resulting in a potential male infertility biomarker.

## 1. Introduction

Infertility is a disease involving both male and female partners with an approximate incidence of 7–15%, and male factor is involved in about half of the cases [1]. Traditionally, the approximation to male infertility issues has relied on basic semen analysis, including sperm concentration, motility, and morphology, but for many couples, these analyses are inconclusive to determine their reproductive potential [2]. To select patients for a treatment with In Vitro Fertilization (IVF) or Intra-Cytoplasmic Sperm Injection (ICSI) semen analysis is used because there is a correlation between morphology of sperm and success with IVF and ICSI. Even so the most effective treatments are those that combine other sperm function tests [3]. Advances in the last decade have shown that sperm DNA integrity may be a good approximation to the sperm natural reproductive capacity and a potential predictor of embryonic development, becoming a suitable marker for male infertility [4,5]. Sperm DNA fragmentation (SDF) can be analyzed using different direct and indirect methodologies, as Terminal deoxynucleotidyl transferase dUTP nick end labeling (TUNEL), Comet assay, Sperm Chromatin Dispersion (SCD) test or Sperm Chromatin Structure Assay (SCSA), among others. The respective advantages and disadvantages of these different methods have been widely reviewed by multiple authors [6,7,8]. Despite the effectiveness of SDF testing for the prediction natural fertility and intra-uterine insemination outcomes, current scientific evidence is has not reached a consensus regarding if SDF is associated to pregnancy rates after ICSI cycles [9], the fact that is probably associated with a lack of standardization of some methodologies among laboratories around the world [9,10,11]. Also, this lack of consensus may also be explained by the oocyte DNA repair capacity, which is related to women’s age [12]. In fact, younger women suffer less implantation failures after IVF cycles [13].

Different intrinsic origins for DNA fragmentation in spermatozoa have been proposed over the years and also reviewed at different works [14,15,16]. First, the abnormal chromatin condensation during spermiogenesis [17], second, the consequence of sperm apoptosis-like [18], and finally, the imbalance of reactive oxygen species and antioxidant capacity [14,19]. Moreover, exogenous factors such as radiations infections, recreational drugs, heat stress, pollution, or the presence of varicocele affectation may also cause oxidative stress, leading to genetic damage that can be produced at different stages of spermatogenesis [20]. Finally, iatrogenic DNA damage caused by sample manipulation prior to performing Assisted Reproductive Technology (ART) is a source of potential breaks [21].

Varicocele is the most common cause of correctable male infertility, with an incidence about 40% of infertile men [22]. The dilatation of the pampiniform plexus is the main affectation of varicocele, causing a blood reflux that leads to an increase of the local temperature at the testis area, all leading to an increase of reactive oxygen species (ROS) and a decrease of antioxidant protection [23]. ROS has been shown to be higher as varicocele grade is worse, leading to a fertility reduction and an increase of DNA damage in affected men [24,25].

After meiosis and spermiogenesis, processes through which spermatogonia lead to differentiated sperm cells, the epididymis is the compartment where sperm maturation takes place, leading to the acquisition of motility and the ability to fertilize an oocyte. Despite the importance of studying these epididymal processes, the assessment of the human epididymis requires invasive procedure. In this sense, the molecular analysis of seminal plasma has been defined as a good approximation for this purpose [26]. It is known that sperm cells only constitute less than 10% of the semen volume, corresponding the remaining 90% to seminal plasma [27]. Seminal plasma (SP) is loaded with proteins that arise from secretions of seminal vesicles (~65% of semen volume), prostate (~25%), testes and epididymis (~10%), and bulbourethral and periurethral glands (~1%) [28]. The main functions of SP components are crucial for the natural reproductive success, as they have a role in regulating the capacitation process, in modulating the immune response and in the interaction and fusion of gametes [29]. Currently, studies analyze SP as a mirror of the estate of accessory glands, testing biochemical parameters like zinc, citric acid, and acid phosphatase in prostates secretions, fructose and prostaglandins in seminal vesicle, and neutral alpha-glycosidase in epididymis [30].

Protein content studies have been performed on SP since 1942 [31]. Recently, the potential of proteomic techniques allow a comprehensive study of the protein profile to compare different estates, enabling the discovery of new and non-invasive biomarkers for diagnostic and treatment procedures [32]. One of the first studies of differential proteomics in SP conducted by Starita-Geribaldi and colleagues [33] compared the proteomic profile of SP samples from fertile individuals, vasectomized and azoospermic individuals, showing potential diagnostic markers of spermatogenesis impairment. Further studies also pursued this aim, by comparing SP protein expression profile of fertile men compared to azoospermic patients [34], by characterizing the protein profile of a healthy individual [35], and by comparing SP from patients with idiopathic fertility and those subjected to assisted reproduction treatments [36]. None of these previous studies took into account the sperm chromatin status for the sample classification prior the analysis of SP and, to the best of our knowledge, only few proteomic studies have been conducted to date has been conducted in samples with a known SDF [37,38].

In order to identify alternative and non-invasive biomarkers that allow diagnosing specific etiologies of male factor infertility, in the present work we conducted a 2D-differential gel electrophoresis (2D-DIGE) approach to detect differential expression of SP proteins among four different groups of samples classified according their clinical status and characterized according semen analysis and DNA integrity.

## 2. Results

### 2.1. Sperm DNA Fragmentation

The analysis of sperm DNA fragmentation and the DNA degraded sperm (DDS) lead to the classification of homogeneous groups of samples: fertile donors (FD), recurrent miscarriage patients (RM), asthenoteratozoospermic patients (ATZ), and asthenoteratozoospermic patients with varicocele (ATZ-VAR); defined in Materials and Methods section. Overall results of each group are described in Table 1.

### 2.2. Analysis of Differentially Expressed Proteins Using 2D-Differential Gel Electrophoresis (2D-DIGE)

**Figure 1:**
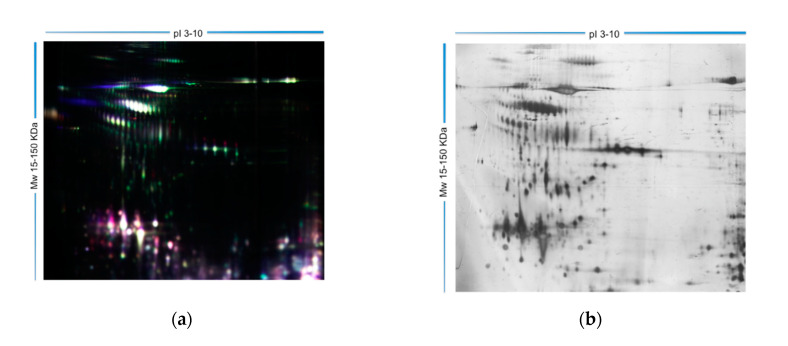
Distribution spots of differentially expressed proteins from seminal plasma samples among fertile and patients group. Representative image in fluorescence difference gel electrophoresis (DIGE) (**a**) and in silver stained (**b**).

The quantitative comparison of seminal plasma proteome profile among all groups analyzed highlighted 96 differentially expressed protein spots (two-fold variation or more in expression, *p* < 0.05). Among them, 26 spots were differential expressed between FD group and all infertile groups, 22 spots were up-regulated and four spots were down-regulated. Eight spots showed up-regulated differential expression in ATZ group respect other groups. Two spots showed down-regulated differential expression between ATZ-VAR group and other groups. RM group showed differential expression in two spots with FD group. A representative 2D-DIGE gel image as example of the gel quality and sample complexity is shown in Figure 1.

### 2.3. Identification of Differentially Expressed Proteins Using Mass Spectrometry (MS)

Out of 96 differentially expressed spots, 42 spots were analyzed by matrix-assisted laser desorption ionization time-of-flight (MALDI-TOF) mass spectrometry (MS) and liquid chromatography with tandem mass spectrometry (LC-MS/MS). From them, 32 spots could be identified, representing 28 different proteins (Table 2). In some cases, spots contained more than one protein and in other cases, different spots represented the same protein, facts that are explained by post-translational modifications. Examples of them are the Prostate-Specific antigen (five spots), Clusterin (six spots), Albumin (four spots), etc. The most pronounced changes in protein levels were detected for Prostate-Specific antigen with a fold change (15-fold, *p* < 0.01), Annexin A3 (9.9-fold each, *p* < 0.01), and Clusterin (4.2-fold each, *p* < 0.01).

### 2.4. Functional Classification of Protein and Protein-Protein Interaction Network

**Figure 2:**
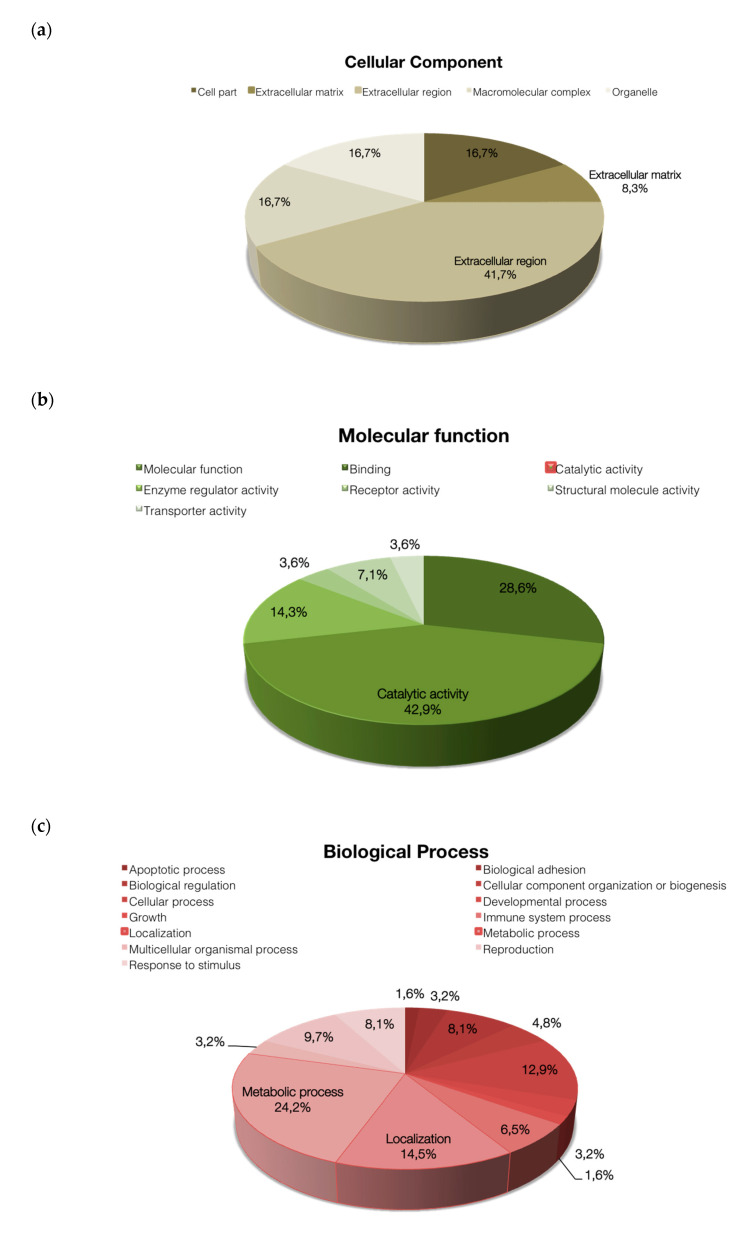
Classification of the differential proteins according to their (**a**) cellular component, (**b**) molecular function, and (**c**) biological process, by using the information available at the Panther Web site.

The functional classification of identified proteins was based on Gene Ontology (GO), using PANTHER 8.0 bioinformatics software platform), which reveals the different functions, processes and cellular localization of the proteins involved. The classification based on cellular localization (Figure 2a) showed that the majority of proteins are presented at extracellular (41.7%) and matrix (8.3%) regions. The classification based on cellular function (Figure 2b) revealed that a large majority of proteins are involved in catalytic activity (42.9%) followed by binding activity (28.6%), enzyme regulator activity (14.3%), structural molecule activity (7.1%), receptor activity (3.6%), and transport activity (3.6%). Regarding biological process classification, proteins are involved in metabolic processes (24.2%), cellular localization (14.5%), cellular processes (12.9%), and reproduction (9.7%) (Figure 2c).

**Figure 3:**
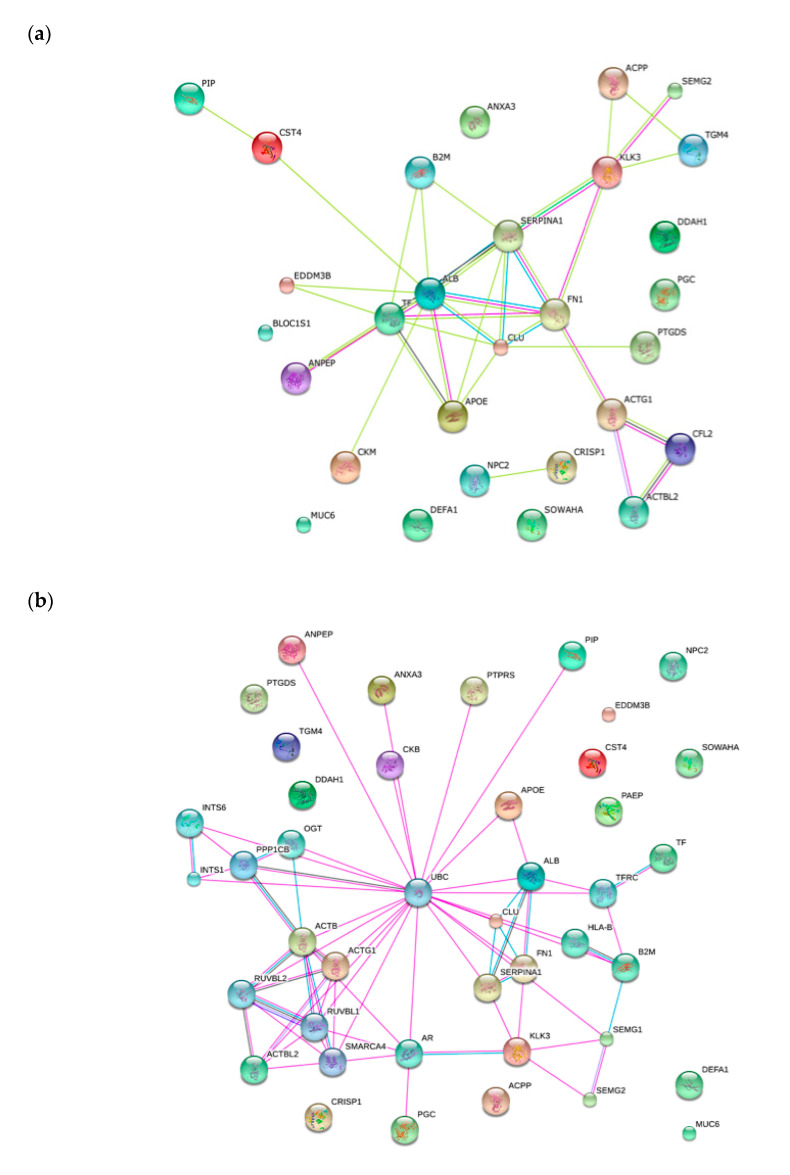
Search Tool for the Retrieval of Interacting Genes/Proteins (STRING) Network nodes among identified proteins (**a**) and in a second shell (**b**).

To create protein-protein interaction network for identified seminal plasma proteins, Search Tool for the Retrieval of Interacting Genes/Proteins (STRING) software was used. The generated network shows the proteins as nodes that are linked through edges. During the search, most of the proteins were clustered into pathways (Figure 3a). Moreover, looked a second level shell, the most highlight is that many of the differential expressed proteins identified in this study interact with Ubiquitin C (Figure 3b).

## 3. Discussion

The use of 2D-DIGE in quantitative proteomics allows the analysis of multiple study groups within the same experimental design. In the present study, this advantage has been used to investigate and compare the seminal plasma protein profile of fertile and infertile males, classified according their clinical status and with well-defined DNA damage and semen analysis parameters. Then, the singularity of the present study remains on this strict definition of homogeneous groups, performed here for the first time (Table 1 and Table 3).

Both motility, morphology, and DNA damage are parameters that help in the diagnosis of the infertile male. In this sense, recent studies using Comet assay provide information about different types of DNA breaks (single- and double-stranded). This distinction is important since our previous studies demonstrated that single stranded DNA damage is caused by oxidative stress and to the capacity of achieving a natural pregnancy, while double stranded DNA breaks are enzymatic and related to a higher risk of recurrent miscarriage [8,39]. In this sense, the DNA damage data obtained for the donors and patients included in the present study fit the specific profiles of single- and double-stranded DNA breaks defined previously [40].

Varicocele is a common cause of male infertility, through the increase of oxidative stress, varicocele patients present affectations in sperm function, leading to altered semen parameters and DNA damage [24]. SCD test allows for to distinguish varicocele patients from infertile patients without varicocele, through the evaluation of the percentage of the total degraded DNA sperm respect total SDF [41]. In the present study, ATZ patients with varicocele also show this profile with high DDS, presenting differences to those patients without varicocele. Surgical varicocelectomy is at the present moment the most effective treatment for varicocele treatment, and different studies have shown that the affected parameters are recovered afterwards [42,43].

In the present study, we took advantage of the proteomics strategy combined to a characterization of homogeneous groups, and we have found 17 proteins with differential expression in FD in comparison to infertile patients the other patients groups analyzed (Table 2).

On one hand, most of these proteins were described before like Prostate-Specific Antigen, Annexin A3, Clusterin, Prostaglandin-H2 D-isomerase, Protein-glutamine gamma-glutamyltransferase 4, Prostatic acid phosphatase, Apolipoprotein E, Glycodelin, semenogelin II, Epidydimal secretory protein E3-beta, Beta-2-microglobulin, CRISP1, and Albumin [37,44,45,46]. PSA is a protein whose expression was found significantly increased in FD in front of the other analyzed patient groups. PSA is known to be responsible for carrying out the liquefaction process in semen, releasing sperm motility active entangled to achieve to reach the egg to fertilize. Also, previous studies found that men with reduced sperm motility had low levels of PSA in the seminal fluid [47,48]. It has also been reported that PSA may have a higher impact on sperm function and fertility due to its role in semenogelins and fibronectin fragmentation [49]. In fact, both proteins are also expressed differentially in the groups studied. Our results, however, are in disagreement with these previous proteomic studies showing a higher expression of PSA in infertile individuals [46,50], fact that could be explained by the presence of different isoforms of the protein [51].

On the other hand, we describe here for the first time, differences in SP (G),*N*(G)-dimethylarginine dimethylaminohydrolase 1 (DDAH1), Serine/threonine-protein phosphatase PP1-beta catalytic subunit, and Ankyrin repeat domain-containing protein SOWAHA. DDAH1 is a protein that acts as a regulator of the generation of nitric oxide that it is involved in a wide range of biological processes [52]. In reproduction, while sustained concentrations of nitric oxide is a necessary compound to achieve capacitation, allowing acrosome reaction and sperm–oocyte interaction [53], it has been reported that high concentrations of nitric oxide play a detrimental effect on sperm motility and sperm function, since the formation of nitrogen radicals and inhibit cell respiration with the consequence of energy loss, and drive protein modifications, membrane lipid peroxidation and DNA fragmentation [54,55].

As expected, differentially expressed proteins between those subjects with normal sperm motility and morphology (FD and RM) and those with abnormal values (ATZ and ATZ-VAR) were found (Table 2): ACTB, KCRB, PEPC, and FN1. In fact, it is well known that these proteins are involved in motility and energy functions, and our results also support their role in the motility and morphology of sperm.

For ATZ samples, an overexpression of the following six proteins has been observed in comparison to other groups: Cystatin-S, Neutrophil defensin 1, Beta-actin, Prolactin-inducible protein (PIP), Alpha-1-antitrypsin, and Serotransferrin (Table 2). The ontology functional analysis revealed that PIP has an endopeptidase activity interacting with other proteins like fibronectin, actin, keratin, myosin, and albumin [56]. Also previous studies showed that they are related to male infertility and to poor sperm quality [57], and found them upregulated in samples with elevated ROS, azoospermia, and asthenoteratozoospermia [34,46,58].

For ATZ-VAR group, Aminopeptidase N (AMPN) was found differential and downregulated protein expression (Table 2). This multifunctional enzyme is involved in sperm regulation and fertility, as previous works have shown that increased AMPN affects sperm motility and early embryo development in mouse [59,60], also acting as a regulatory factor in angiogenesis in the female reproductive system [61].

The fact that most of the proteins analyzed here are involved in metabolic processes and catalytic function (Figure 2) suggests that SDF from the analyzed groups is mostly caused by oxidative damage. It is well known that non-fertile sperm present deregulation of proteins involved in metabolism, energy production, leading to high ROS, causing the aforementioned detrimental effects [38,62]. Also, it is noteworthy that post-translational modifications occur before proteins are poured into seminal fluid, since we have found similar proteins at different spots. These post-translational modifications, including ubiquitination, phosphorylation, acetylation, sumoylation, and others, may have an important role in sperm due to its transcriptional and traductional inactivity, thus helping in the regulation protein stability and activity both in physiological and pathological states [63]. Interactions among differential proteins identified in the present study provide a substantial confirmation that ubiquitin C might indeed be involved in sperm fertility regulation (Figure 3a,b). Ubiquitin is known to play a role in regulating multiple cellular pathways by linking a variable number of ubiquitin chains at different protein positions in targeted proteins [64]. In reproduction, it has been described to have a role in fertilization by marking defective sperm, remodeling plasma membrane in capacitation, in acrosome reaction, in promoting sperm–oocyte interaction and in paternal mitochondrial inheritance [65]. Also, studies showed the presence of ubiquitinated sperm cells in samples with DNA fragmentation, suggesting a role in semen quality control [66,67].

In summary, the present study identifies for the first time a differential protein expression in SP in different groups homogeneously defined by pathology, semen analysis and DNA fragmentation. The proteomic data by 2D-DIGE confirm the different expression of 28 proteins as potential biomarkers of infertility. Overall, the present data suggest that major proteins are involved in energy metabolism, catalytic function and have an extracellular location. Indeed, the present results strongly support the interaction between the differential proteins and ubiquitin C role in degradation of those that are defectives. Further experiments might explore the use of the detected proteins as potential prognostic markers of infertility and ART outcomes.

## 4. Materials and Methods

### 4.1. Semen Samples and Sperm DNA Fragmentation

#### 4.1.1. Samples and Study Design

Semen samples have been collected from six fertile donors and 18 infertile patients. All samples were collected by masturbation after 2–5 days of recommended abstinence. Written informed consent was obtained for all patients and the hospital ethical committee approved the present study on 2 September 2017 with registration number 2017902 and all methods were carried out in accordance with the Code of Ethics of the World Medical Association (Declaration of Helsinki) guidelines and regulations. The inclusion criteria for the study enrollment was to belong to one of the clinical groups defined for the study: Fertile donors, recurrent miscarriage patients with at least 2 miscarriages, and infertile patients without or with varicocele. Infertility has been defined by the WHO as the inability to achieve a clinical pregnancy in 12 months of unprotected relationships. No exclusive criteria were defined regarding smoking, drinking, the use of recreational drugs, etc.

After the collection of semen samples, seminal plasma was separated from sperm fraction and kept until sample groups were established. Basic analysis of sperm, including concentration, motility, and morphology, was performed following WHO guidelines and thresholds. Sperm DNA damage analysis through SCD, alkaline Comet and neutral Comet were performed following the protocols described below with the aim of defining homogeneous groups of samples that would be subjected to proteomic studies.

Then, SP samples were categorized in four groups according pathology, basic sperm analysis and DNA status, taking into account the threshold values summarized in Table 3:

1. Fertile Donors (FD), including samples from six fertile donors who presented normal semen analysis, low DNA degraded sperm (DDS), low SDF by alkaline and neutral Comet assay.
2. Recurrent Miscarriage (RM), including samples from six recurrent miscarriage patients without female factor, who presented normal semen analysis (sperm count, motility and morphology), low DDS, low SDF by alkaline and high SDF by neutral Comet assay.
3. Asthenoteratozoospermic infertile patients without varicocele (ATZ), including samples from six ATZ patients, with low DDS, high SDF by alkaline and neutral Comet assay.
4. Asthenoteratozoospermic infertile patients with varicocele (ATZ-VAR), including samples from six ATZ patients with varicocele, with high values of DDS and SDF for both Comet assays.

#### 4.1.2. Semen Collection, Basic Semen Analysis, and Cryopreservation

After liquefaction, routine semen analysis was performed according to WHO (2010) and, subsequently, the semen was divided into two aliquots; 500 µL of unprocessed semen sample was cryopreserved with Test yolk buffer (14% glycerol, 30% egg yolk, 1.98% glucose, 1.72% sodium citrate), and 500 µL was centrifuged at 13,300 g for 20 min at 4 °C, the resulting supernatant being aspirated and stored at −80 °C until use for 2D-DIGE.

#### 4.1.3. DNA Integrity Tests: SCD, Alkaline Comet and Neutral Comet

SCD and Comet assays have a common first steps that comprise sperm thawing for 30 s at 37 °C, washing three times in PBS (phosphate-buffered saline), adjusting the concentration at 10^6^ sperm/mL, mixing sperm sample with low melting point agarose 1% (Sigma Aldrich; St Louis, MO, USA) at 1:2 ratio, and jellifying the mixture in a pre-treated slide for gel adhesion at 4 °C for 5 min.

After, the coverslips were carefully removed and alkaline and neutral Comet assays and SCD test were performed according the protocols described before for Comet assay [40], and in the manufacturer’s instruction for SCD.

The assessment of fragmented and non-fragmented sperm cells were conducted by the assessment of 500 sperm previously dyed with antifade DAPI Slowfade Gold (Invitrogen; Eugene, OR, USA) and under the epifluorescence microscopy. For Comet assay, the classification of fragmented or non-fragmented sperm and the cut-off values for alkaline and neutral Comet assay were described by our research group [40]. For the assessment of the DNA degradation index (DDS), SCD test was performed according to the manufacturers’ instructions of the Halosperm Kit (Halotech DNA; Madrid, Spain). Degraded sperm (DDS), besides not having haloes, were characterized by the presence of a faint and/or nonuniformly stained chromatin core. DDS index was calculated as the proportion of degraded sperm in the whole population of sperm with fragmented DNA, the cut-off value was 0.33 or above [41].

### 4.2. Proteomic Analysis of Seminal Plasma

#### 4.2.1. Sample Preparation for 2-D DIGE

SP proteins were precipitated by 2-DE Clean-Up kit (GE Healthcare, Chicago, IL, USA), resuspended in a buffer containing 7 M urea, 2 M thiourea, 40 mM Tris, 4% *w/v* CHAPS, pH 8.5 and finally stored at −80 °C until use. A RC DC Protein Assay Kit (Bio-Rad, Hercules, CA, USA) was used to determine protein concentration of samples.

#### 4.2.2. Protein Labeling with CyDye DIGE

Protein labeling was performed as previously described [69]. A pairwise comparison was performed among the four groups of study. In brief, each group were differentially labeled at a concentration of 400 pmol of CyDye DIGE fluors (Cy3 or Cy5) per 50 μg of protein for 30 min in the dark at 4 °C. To eliminate variations between gels an internal standard composed by a pool of all samples (fertile and infertile patients) and labeled with Cy2 was used. Finally, the same amounts of Cy3, Cy5, and the standard with Cy2 were mixed to run on the gels.

#### 4.2.3. Two-Dimensional Gel Electrophoresis

Immobiline Dry strips 24 cm, pH 3–10 (GE Healthcare) were rehydrated overnight in 450 μL of buffer (7 M urea, 2 M thiourea, 2% *w/v* CHAPS, 0.5% *v/v* IPG pH 3–10 buffer, 50 mM DTT, 1.2% *v/v* DeStreak (Sigma)) and were loaded with one hundred and fifty micrograms of protein. The first dimension of isoelectric focusing was run on an IPGphor EF System (GE Healthcare, Chicago, IL, USA). Optimal protein focusing was achieved by starting at 300 V for 5 h, followed by a two steps at 1000 V for 6 h and 8000 V for 3 h. in the gradient and focusing from 8000 to 56,000 V/h. Strips were stored at −80 °C. Before second dimension, the strips were thawed and equilibrated in two steps for 15 min each, first buffer (50 mM Tris-HCl, 30% *v/v* glycerol, 6 M urea, 10 g/L DTT, 2% *w/v* SDS, and 0.01% *w/v* bromophenol blue, pH 8.8), the second also contained 2.5% iodoacetamide. The second dimension was performed by 12% polyacrylamide gels. Strips were loaded onto the gels and sealed with a solution of 0.5% agarose (*w/v*) containing a trace of bromophenol blue. The gels were run on the Ettan DALT VI (GE Healthcare, Chicago, IL, USA) at 2,5 W/gel for 30 min followed by 12 W/gel till the bromophenol blue dye front reached the bottom of the gel at 20 °C.

All gels were scanned using a Typhoon image scanner 9410 (GE Healthcare, Chicago, IL, USA) at three different emission/excitation wavelengths, namely, 488/520, 532/580, or 633/670 nm and silver stained was carried out using a standard protocol for MS analysis. After scanning, the 2-D DIGE images were analyzed with the software Progenesis SameSpot (Nonlinear Dynamics, Newcastle, UK). Volumes of individual spots were normalized against the total volume of all the spots in the gel. Spot volume ratio change with *p* < 0.05 and having an absolute fold change greater than two were criteria for MS identification of abundance differences.

#### 4.2.4. Spot Picking and In-Gel Digestion for MS Analysis

MS was performed as described previously [69]. Gels were stained with silver and then the spots selected for analyses were manually excised and digested using an automatic device (DigestPro MS, Intavis). The processing involved reduction with dithiothreitol (DTT), derivatization with iodoacetamide (IAA), and enzymatic digestion with trypsin (37 °C, 8 h). The resulting peptide mixture was spotted on a MALDI plate and analyzed using a MALDI-TOF/TOF MS (ABI-Sciex 4800). For each sample one MS spectra was obtained. The MALDI-TOF spectra were interpreted by database search (Mascot, Matrix Science) with a significance threshold of the Molecular Weight Search (MOWSE) score of *p* < 0.05. All identifications were manually validated. The database used for identification was SwissProt restricted to human proteins.

Each unidentified spot was reanalyzed by LC-MS/MS. Proteins were dissolved using a buffer containing urea and DTT. The extract was derivatized with iodoacetamide and digested according to internal protocols. The tryptic extracts were analyzed by LC-MS/MS in data-dependent mode. The MS system used was a Velos LTQ (ThermoFisher, Waltham, MA, USA) equipped with a microESI ion source. The tryptic extracts were diluted to 20 µL with 5% methanol with 1% formic acid and loaded into a chromatographic system (Agilent Technologies, Santa Clara, CA, USA). The Velos LTQ was operated in the positive ion mode with a spray voltage of 2 kV. The scan range for full scans was *m*/*z* 450–2000.

#### 4.2.5. Protein Identification

The obtained mass spectra were subjected to a Mascot search engine. Probability-based protein identification was performed by searching sequence databases using mass spectrometry data with 0.8 Da peptide tolerance, two miss-cleavages, carbamidomethylation and methionine oxidation as variable modifications. Protein annotation was performed by using the Uniprot database.

#### 4.2.6. Functional Enrichment Analysis

Analysis by PANTHER database (Protein Analysis Through Evolutionary Relationships, http://pantherdb.org) was used to determine the percentage of proteins from our dataset involved in the “molecular function”, “biological process” and “cellular component”. The obtained PANTHER data was further analyzed, and graphs were prepared using MS Excel 2007.

STRING database (version 8.3; http://string.embl.de/) was used to identify the protein-protein interaction network to show the interactions of identified proteins with a specific group of molecules.

## Figures and Tables

**Table 1:** Percentage of spermatozoa showing DNA fragmentation assessed by the Comet assay and Sperm Chromatin Dispersion (SCD) test (mean ± standard deviation).

Group and Pathology	SDF (Alkaline Comet)	SDF (Neutral Comet)	DDS (SCD Test)
Group FD (*n* = 6)	23.53 ± 10.46	12.36 ± 4.31	<0.33
Group RM (*n* = 6)	36.73 ± 16.42	62.74 ± 12.47	<0.33
Group ATZ (*n* = 6)	67.29 ± 12.66	74.29 ± 5.91	<0.33
Group ATZ-VAR (*n* = 6)	71.22 ± 5.91	86.59 ± 11.83	>0.33

**Table 2:** Proteins with differential expression in seminal plasma from fertile donor group vs. patient groups analyzed.

Group and Pathology	Protein Name	Symbol	Uniprot Accession Number	Fold-Change		
				RM	ATZ	ATZ-VAR
Group 1: FD (*n* = 6)	*N*(G),*N*(G)-dimethylarginine dimethylaminohydrolase 1	DDAH1_HUMAN	O94760	2.0	2.7	2.2
	Annexin A3	ANXA3_HUMAN	P12429	0.022	0.946	0.021
	Clusterin	CLUS_HUMAN	P10909	13.1	11.7	2.0
	Prostaglandin-H2 D-isomerase	PTGDS_HUMAN	P41222	2.3	2.6	2.6
	Semenogelin-1	SEMG1_HUMAN	P04279	2.8	1.5	2.7
	Beta-2-microglobulin	B2MG_HUMAN	Q91966	3.7	2.5	2.5
	Prostate-specific antigen	KLK3_HUMAN	P07288	15.0	11.4	2.2
	Protein-glutamine gamma-glutamyltransferase 4	TGM4_HUMAN	P49221	3.8	4.1	4.4
	cDNA FLJ78262, highly similar to Homo sapiens semenogelin II (SEMG2), mRNA	A8K6Z6_HUMAN	A8K6Z6	4.6	5.0	3.9
	cDNA FLJ75803, highly similar to Homo sapiens cysteine-rich secretory protein 1 (CRISP1), transcript variant 1, mRNA	A8K8Y2_HUMAN	A8K8Y2	1.9	2.5	1.8
	Prostatic acid phosphatase	PPAP_HUMAN	P15309	2.0	2.7	2.2
	Ankyrin repeat domain-containing protein SOWAHA	SWAHA_HUMAN	Q2M3V2	3.7	2.5	2.5
	Serine/threonine-protein phosphatase PP1-beta catalytic subunit	PP1B_HUMAN	P62140	2.0	2.7	2.2
	Epididymal secretory protein E3-beta	EP3B_HUMAN	P56851	2.2	2.2	2.6
	Albumin	F6KPG5_HUMAN	F6KPG5	4.2	2.7	4.7
	Apolipoprotein E	Apolipoprotein E (Fragment)	D9ZB55	1.9	2.5	1.8
	cDNA, FLJ92074, highly similar to Homo sapiens progestagen-associated endometrial protein (placental protein 14, pregnancy-associated endometrial alpha-2-globulin, alpha uterine protein) (PAEP), mRNA	B2R4F9_HUMAN	B2R4F9	5.4	3.6	2.9
				**RM**	**ATZ**	**ATZ-VAR**
Group 2: normal semen analysis (FD and RM) vs. abnormal semen analysis (ATZ and ATZ-VAR)	Creatine kinase B-type	KCRB_HUMAN	P02787	1.3	2.3	2.2
	Gastrisin	PEPC_HUMAN	P20142	1.7	2.0	2.1
	Actin, cytoplasmic 1	ACTB_HUMAN	P60709	1.0	1.8	1.9
	Fibronectin 1 (FN1)	Q9UQS6_HUMAN	Q9UQS6	1.2	2.0	2.0
				**RM**	**ATZ**	**FD**
Group 3: ATZ vs. other groups	Serotransferrin	TRFE_HUMAN	P02787	1.3	1.1	2.5
	Prolactin-inducible protein	PIP_HUMAN	P12273	1.3	1.4	2.2
	Beta-actin	F1BXA6_HUMAN	F1BXA6	1.3	1.4	2.2
	Neutrophil defensin 1	DEF1_HUMAN	P59665	1.3	1.4	2.2
	Cystatin-S	CYTS_HUMAN	P01036	2.4	2.7	4.4
	Alpha-1-antitrypsin	A1AT_HUMAN	P01009	1.5	1.9	2.2
				**RM**	**ATZ**	**FD**
Group 4: ATZ-VAR vs. other groups	Aminopeptidase N	AMPN_HUMAN	P15144	3.1	2.5	2.0

**Table 3:** Characteristics of patients for 2D-differential gel electrophoresis (2D-DIGE) analysis selected by pathology, DNA degraded sperm (DDS), and Sperm DNA fragmentation (SDF) values ^1^, measured by SCD test and Comet assay, respectively.

Group and Pathology	DDS (SCD Test)	SDF (Alkaline Comet)	SDF (Neutral Comet)
FD (*n* = 6)	<0.33	<45%	<50%
RM (*n* = 6)	<0.33	<45%	>50%
ATZ (*n* = 6)	<0.33	>45%	>50%
ATZ-VAR (*n* = 6)	>0.33	>45%	>50%

^1^ For DDS, the cut-off value for varicocele patients is 0.30 [41]. The cut-off value for alkaline Comet assay is 45% of SDF and for neutral comet assay is 50% of SDF [68].

## Abbreviations

2D-DIGE	2D-Differential Gel Electrophoresis
SP	Seminal Plasma
SDF	Sperm DNA Fragmentation
FD	Fertile Donors
ATZ	Asthenoteratozoospermic Group
ATZ-VAR	Asthenoteratozoospermic with Varicocele Group
RM	Recurrent Miscarriage
SCD	Sperm Chromatin Dispersion
MS	Mass Spectrometry
DDS	Degraded DNA Sperm
ART	Assisted Reproductive Technology
ICSI	Intra-Cytoplasmic Sperm Injection
